# Development of FamTAM Intervention for Speech and Language Pathologists and Family Members

**DOI:** 10.3390/bs16091571

**Published:** 2026-09-04

**Authors:** Sarah Nathel Douglas, Hedda Meadan, Sarah Dunkel-Jackson, Atikah Bagawan, Adriana Kaori Terol, Alexandra Reilly

**Affiliations:** 1 Human Development and Family Studies, Michigan State University, East Lansing, MI 48824, USA; dunkelsa@msu.edu; 2 Department of Special Education and Child Development, University of North Carolina at Charlotte, Charlotte, NC 28223, USA; hedda.meadan@charlotte.edu (H.M.); reillya2@cofc.edu (A.R.); 3 Department of Psychology, Islamic University of Riau, Pekanbaru 28284, Indonesia; atikahaira@psy.uir.ac.id; 4 MIND Institute, University of California Davis, Sacramento, CA 95817, USA; aterol@health.ucdavis.edu

**Keywords:** FamTAM Intervention, augmentative and alternative communication, aided language modeling, interviews, intervention development, coaching

## Abstract

Children with complex communication needs, including approximately one-third of children with autism, use augmentative and alternative communication (AAC) to meet their daily communication needs. AAC includes gestures, sign language, picture symbols, and computerized systems to augment or replace speech. Family support and training are essential for effective AAC implementation, but few interventions exist. We describe the iterative development of the FamTAM Intervention—an aided language modeling training and coaching intervention designed to support caregiver implementation and their school-based speech language pathologists (SLPs) preparedness to provide family coaching. We detail the iterative process, including refining the training content and delivery, expert reviews, and interviews with SLPs and caregivers. We also conducted interviews with participants to assess the clarity, feasibility, acceptability, and usability of the intervention. Our findings indicate that participants viewed the FamTAM Intervention as engaging, practical, and aligned with evidence-based AAC practices, while also identifying areas for further refinement.

## 1. Introduction

Individuals with complex communication needs often benefit from the use of augmentative and alternative communication (AAC) systems, including no-tech (e.g., gestures, facial expressions), low-tech (e.g., exchanging pictures, spelling words by pointing to letters), and high-tech (e.g., tablet with speech generating software) systems (ASHA, n.d.). Engaging family members in the use of their child’s AAC system is an essential part of the child’s communication development (Coburn et al., 2021). Indeed, evidence suggests that children’s communication development is influenced by training provided to families to support their child’s communication (Roberts & Kaiser, 2011; Smith et al., 2016). Children experience the best outcomes when family members are well-trained and highly engaged (Seligman & Darling, 2017). However, families are rarely provided with sufficient training to support the use of AAC (Kent-Walsh et al., 2015). Training a primary caregiver to support the child’s communication can benefit the child who uses AAC (Roberts & Kaiser, 2011) and help the primary caregiver and other family members learn to communicate with the child in more responsive ways. This carryover from one caregiver to another can help develop the child’s communication throughout the day and across activities and caregivers.

Most often, communication interventions are delivered by professionals (e.g., speech and language pathologists, behavior therapists, teachers), which may result in positive outcomes on some measures of child communication, but not all (Roberts & Kaiser, 2011). For example, in their meta-analysis, Roberts and Kaiser (2011) noted that children benefit more from parent-implemented communication interventions compared to those who did not engage in intervention across several language constructs (e.g., receptive and expressive language, receptive and expressive vocabulary, expressive morphosyntax, rate of communication). However, effect sizes were smaller when outcomes were compared between parent-implemented and therapist-implemented interventions, finding no difference between parent- and therapist- implemented interventions across several constructs. Although stronger measures of (a) child communication (e.g., direct-observation instead of only parent-report) and (b) treatment integrity are needed (Roberts & Kaiser, 2011), these data suggest that caregiver-implemented interventions can supplement therapist-implemented interventions to further support their child’s communication. By addressing methodological gaps (i.e., direct measurement, treatment integrity), researchers can identify which specific interventions are most effectively implemented by professionals and which can be implemented by parents and other caregivers to promote the child’s communication development.

Speech and language pathologists (SLP) often lead the selection of appropriate AAC systems and AAC implementation with families and educational teams (Douglas et al., under review-b). Partnering with families requires that professionals establish rapport by being open minded, identifying a shared vision, and honoring the priorities, interests, needs, goals, and experiences of family members (Bagawan et al., in press; Smith et al., 2016; Soto, 2012; Woods et al., 2011). Professionals must be reciprocal in their approach, respectful of cultural beliefs and values, and engage in active listening as well as culturally responsive communication (ABAI, n.d.; BACB, 2020; Soto, 2012). Adapting to the needs of the family in terms of communication goals, interventions to support communication, and the format in which they receive coordinated support (ABAI, n.d.; Douglas et al., 2022; Kummerer, 2012; Soto, 2012) can empower the family to implement specific strategies that encourage child communication.

Aided language modeling (ALM) is an approach that has been shown to support language learning, AAC use (Pope et al., 2025), and social interaction (Biggs et al., 2018b). A systematic review that included 48 studies found ALM to be effective at improving expressive communication outcomes of children of various ages and disabilities (Biggs et al., 2018b). The benefit of ALM has been found both in schools and home settings (Douglas et al., 2021, 2023; Biggs et al., 2018b; Quinn et al., 2020). In the school setting, recent research has found an improvement in symbolic communication and participation by young children with down syndrome during small group dialogic reading (Quinn et al., 2020). ALM has also been taught to peers of children who use AAC (Biggs et al., 2018a). Preschool peers who were trained to model AAC showed increased communication towards their friends who used AAC (Thiemann-Bourque et al., 2017). In turn, the child who used AAC also showed improved communication reciprocity and engagement with their peers.

**Figure 1:**
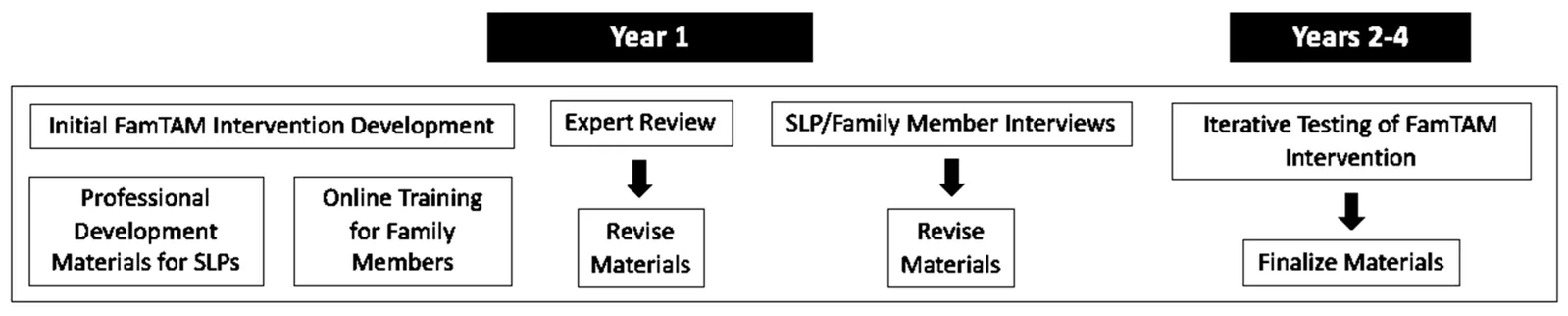
FamTAM Intervention development process.

ALM has also been used in home settings. Douglas et al. (2021) trained and coached four family members of a child through telepractice on the implementation of ALM and found that, with coaching and feedback, family members increased the fidelity of ALM use during daily routines. Consequently, the child also began to use their AAC system more independently. A follow-up study was also conducted, where the main caregiver (mom) of a child who used AAC trained and coached ALM to four other family members (Douglas et al., 2023). Results showed that family members were able to use the ALM strategy with high fidelity, and there was a modest increase in the child’s use of AAC. These studies show positive effects when the child’s family is given proper training and coaching on the use of ALM. Additionally, the cascading intervention model has been shown to build family capacity and increase sustainable ALM use within the family as well as create communication opportunities for the child. Aside from these studies, however, few studies include family members implementing ALM strategies, especially when training is tailored to the child’s individual needs. Additionally, there is no existing literature where school-based SLPs have provided support to family members while implementing ALM. To fill this gap in the literature, we developed the FamTAM (family training in aided language modeling) intervention, which includes online professional development for school-based SLPs to coach family members to support intervention implementation and online training for family members of children who use aided AAC. The purpose of this article is to describe the iterative process to develop the FamTAM Intervention. We detail each step of the iterative process we used next, including initial intervention (a) development; (b) expert review; and (c) SLP/family member interviews (see Figure 1).

## 2. FamTAM Intervention Development: An Iterative Process

The iterative development process used to create the FamTAM Intervention was informed by the person-based approach, which offers a practical, user-centered framework for intervention development by grounding the design process in a detailed understanding of the target population’s experiences, needs, and preferences (Yardley et al., 2015). We selected the person-based approach because it allowed us to explore how individuals perceived and engaged with the intervention in real-world settings. The person-based approach also helped ensure that the final FamTAM Intervention would be acceptable, meaningful, and feasible for the target population (Holt et al., 2025). A person-based approach is informed by both quantitative (e.g., questionnaire) and qualitative methods (e.g., interviews, focus groups) throughout the development process to support the iterative refinement of intervention components. By examining how individuals perceive and engage with an intervention, researchers can identify barriers to implementation and make adjustments that enhance acceptability, feasibility, and relevance in real-world contexts. Consequently, interventions developed using the person-based approach are more likely to be meaningful to users and to support sustained engagement and behavior change, particularly in complex or context-sensitive settings (Bradbury et al., 2019; Yardley et al., 2015).

Prior to starting the research project, we obtained internal review board (IRB) approval from our educational institutions. Given the qualitative aspects of the development process, we engaged in several strategies to ensure the trustworthiness of our findings, including a team approach to data analysis using investigator triangulation (Denzin, 2017), field notes, and member checks (Lincoln & Guba, 1985). These processes, in addition to weekly team meetings, were used to strengthen the credibility, dependability, and confirmability of our findings during the development process. Our team was all women, four of whom identified as White, one as Hispanic, and one as Asian. All team members had earned or were pursuing a PhD, with expertise in behavior analysis, early childhood education, special education, speech and language pathology, and AAC. All team members had experience implementing interventions with family members and professionals. We were mindful during the development process to engage in regular reflection during our weekly team meetings to minimize our own biases.

## 3. Initial Intervention Development

The initial intervention development process included the creation of materials for (a) online professional development for school-based SLPs to coach caregivers to support intervention implementation; and (b) online training for caregivers. Intervention materials were hosted on a Canvas digital learning platform (Instructure, 2024). FamTAM materials were created using instructional components recommended within communication partner training programs (Kent-Walsh & McNaughton, 2005; Kent-Walsh et al., 2015) and those recommended for adult learners (Lim, 2004). FamTAM components included an overview, description, and demonstration of the ALM strategies as well as feedback during knowledge checks. Online materials were available asynchronously to ensure flexibility for busy SLPs and families. Additionally, the training included embedded questions to check the recipients’ understanding of the content. Instructional methods were utilized within the online training to ensure participants were actively engaged in the learning process, interacted with materials, and mastered content (Lebel et al., 2005).

### 3.1. Professional Development Materials for SLPs

**Figure 2:**
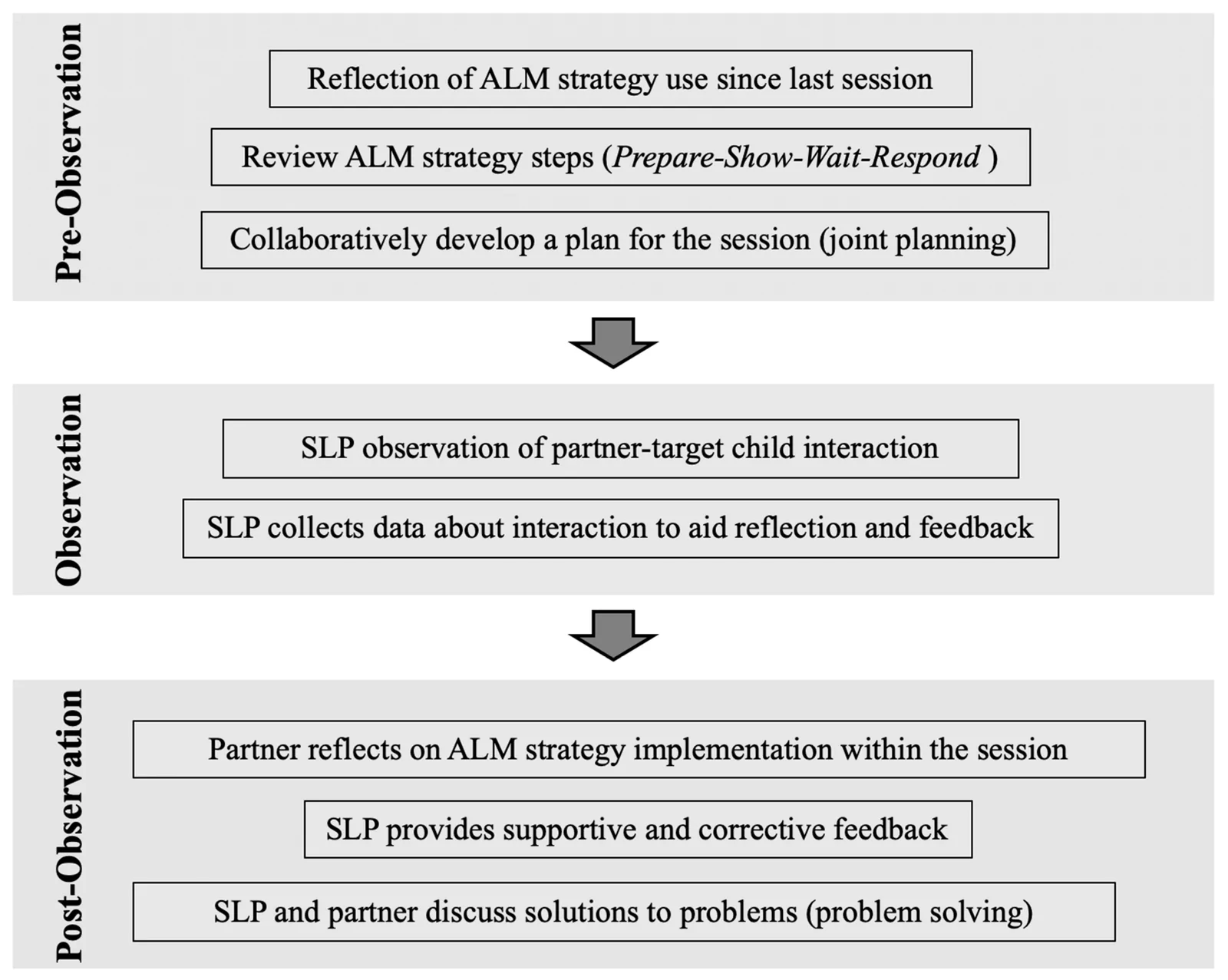
Coaching flowchart.

First, we began creating professional development materials for school-based SLPs. The materials for SLPs were revised from our previous coaching materials with family members (Douglas et al., 2021, 2023). Training materials included content related to ALM implementation as well as content related to partnering with and coaching family members. The materials included an introduction to the FamTAM Intervention and its structure, a FamTAM Intervention manual, content about family-centered practices and family/professional partnerships, coaching practices, the Prepare–Show–Wait–Respond ALM strategy, and data-based decision making. The content and instructional components for SLPs are detailed in Table 1. The coaching protocol included three parts: (a) pre-observation reflection and planning, (b) observation of the family member-child dyad, and (c) post-observation reflection and feedback (Meadan et al., 2016, 2020, 2022; see Figure 2). Coaching sessions were designed to be completed in approximately 20 min.

### 3.2. Online Training Materials for Family Members

We began developing online training for family members using training materials from our previous ALM interventions with families (Douglas et al., 2021, 2023). These materials were previously used with two families, so our first round of revisions were based on feedback from families in those studies and adjustments to improve outcomes for family members based on past participant performance (e.g., additional video models and examples within training). At this stage, we also added content to ensure the intervention was culturally relevant for families from a wide variety of backgrounds (i.e., new images, inclusive language, captioning for videos). The materials primarily focused on the Prepare–Show–Wait–Respond ALM strategy, a communication partner strategy to support AAC use and aided language modeling. We also designed the training to include follow-up coaching from SLPs. These materials are detailed within the SLP professional development. The content and instructional components for family members are detailed in Table 1.

## 4. Expert Review

Aligned with the intervention planning stage in the person-based approach (Yardley et al., 2015), we conducted an expert review of the developed materials. Expert review was done to ensure the content was clear, included sufficient examples, and aligned with best practices and recommendations in the literature. We also asked the expert reviewers to provide feedback about the representation of diverse populations in the materials, the check for understanding questions, and the resources that were provided.

### 4.1. Expert Reviewers

Because our goal was to develop an intervention grounded in research and best practices, our expert reviewers were identified during the grant funding process for the project. Experts were selected to ensure adequate knowledge in areas relevant to our intervention. They included four women who all held doctoral degrees and were faculty at higher education institutions in the field of special education or speech language pathology. Our expert reviewers had both practical and research expertise in the areas of AAC intervention, professional development, culturally relevant practices, school-based SLPs, telepractice, partnering with families, and ALM. One expert reviewer identified as Hispanic while the other three identified as White.

### 4.2. Expert Review Procedures

After the initial development was complete, the FamTAM Intervention materials were shared with our expert reviewers for feedback. Expert reviewers completed a questionnaire about the content, format, procedures, and overall impressions of all FamTAM Intervention materials. The questionnaire included 15 Likert-scaled items (from 1 strongly disagree to 4 strongly agree) for the three sections of training, as well as four open-ended responses for each section of the training. Likert-scaled items from our expert reviewers were analyzed descriptively, while open-ended responses were analyzed using a qualitative content analysis approach (Saldaña, 2021). This process was used to understand areas that needed clarity, improvement, or adjustment. Given the limited open-ended responses, content analysis focused primarily on any positive feedback and adjustments that were needed. This process was led by the first author, but all authors reviewed the open-ended questions and met to discuss the adjustments and changes needed within the training. Each open-ended comment in the questionnaire that indicated something positive about the training or an area needing adjustment was categorized (e.g., content aligned well with the literature; add details about the included resources). Expert reviewers were provided with a stipend for the time they devoted to providing feedback. The expert review materials are provided in the Supplementary Materials (S1).

### 4.3. Expert Review Findings

Results from our Likert-scaled items and content analyses of expert reviewers provided insights into relevant adjustments needed to enhance the FamTAM Intervention. Overall, the expert reviewers reported that the FamTAM materials were appropriate. One expert reviewer said: “Terrific content! Presented in clear and easy to follow steps.” Specifically, all expert reviewers felt that the content, across the three sections, aligned well with the literature and best practice (M = 3.6; range 3–4), the videos were helpful (M = 3.8; range 3–4), and checks for understanding within the digital learning platform were appropriate (M = 3.4; range 3–4). An expert reviewer said: “Video examples are well done and add nicely to the participant’s learning experience.” Although all expert reviewers agreed that the content aligned well with the literature and best practice, one recommendation was provided within open-ended questions: “When suggesting ‘wait time’ I would recommend being flexible… 5 s may not be enough for [children with motor impairments] to access their desired words.”

Although most reviews were generally positive for the content and procedures, suggested improvements were provided for the examples (M = 3.3; range 2–4), resources (M = 3.4; range 2–4), and representing of diverse populations (M = 3.3; range 2–4). In the area of providing sufficient examples, one expert reviewer expressed that the models we provided for SLPs could be improved: “SLP models may benefit from a little more depth/clarification or some additional practical examples.” In the area of resources, three reviewers mostly agreed or strongly agreed that they were sufficient. Only one expert reviewer felt that improvements should be made to the family member resources: “It would be helpful to have a bit more information about each suggested resource…an annotated bibliography.” One expert reviewer also had suggestions to improve the representations of diverse populations: “Cultural diversity could be more extensive…go beyond diversity in photographs.” The reviewers also provided suggestions for formatting (e.g., adjustment to fonts, reducing text on slides, including additional graphics).

### 4.4. Adjustments Based on Expert Review

After expert review was complete, adjustments were made to the materials based on their feedback. Table 2 provides examples recommendations, illustrative quotes, and examples of adjustments made. Feedback included requests to explain the importance of flexibility regarding wait time length with a range to support the child and justification from the existing research (see Sun et al., 2023). We also included additional examples, resources, and expanded representations of diversity. After suggestions were incorporated into iteration 1 of the FamTAM materials based on the expert review, we conducted interviews with SLPs and families.

## 5. Interviews

Online individual and group interviews (Krathwohl, 2009) were conducted with SLPs and family members of children who use aided AAC. We utilized the Consolidated Criteria for Reporting Qualitative Research (COREQ) guidelines to support comprehensive reporting of the interviews from this project (Tong et al., 2007). Participants for interviews were recruited through our professional networks (including our expert reviewers) as well as postings within social media groups that focused on AAC, and were geared towards family members and SLPs. All interested participants completed a screening form to confirm their authenticity as well as eligibility for participation in the study. SLP participants were eligible if they supported children who use AAC ages 4–10 years in school-based settings. Family members were eligible to participate if they had a child who used AAC ages 4–10 years. Although we had 28 SLPs and 40 family members complete the screening form, 18 were identified as non-genuine during the screening process (e.g., duplicate IP addresses, inconsistent responses, or flagged as foreign responses with geolocation), and 14 did not meet the inclusion criteria based on the responses in their questionnaire (7 SLPs, 7 family members). Others who did not end up participating either did not respond to emails to schedule the interview (3 SLPs; 7 family members), could not attend the scheduled interview (3 SLPs; 2 family members), or did not attend at the scheduled interview time (2 SLPs; 3 family members). Participants across our individual and group interviews represented 10 different states and all five regions of the United States. Interviews were conducted by grouping participants by their roles, with separate interviews for SLPs (*n* = 8) and family members (*n* = 8).

### 5.1. Interview Participants

Four separate interviews were conducted with SLP participants. One group interview was conducted with four SLPs, and due to scheduling conflicts, the remaining four SLPs were interviewed one at a time. SLP interviews were 32 min on average (range = 13–62 min) with the group interview being longer than individual interviews. Participants in the SLP interviews included seven women and one non-binary participant, all of whom were SLPs in educational settings and had Master’s degrees. Six SLP participants identified as White, one identified as Asian, and one identified as Hispanic. Their mean age was 40 years old (range = 24–59), and mean years of experience as an SLP was 14 (range = 1–30). They worked across rural, town, suburban, and urban locations. Three SLPs were fluent in a language other than English (i.e., Tagalog, American Sign Language, Spanish). All supported children who used AAC in school settings with a variety of disabilities (e.g., autism, speech language impairment, intellectual disability, multiple disabilities, hearing impairment, visual impairment, traumatic brain injury).

Two separate group interviews were conducted with family members. Based on availability, the first group interview included six family members and the second group interview included two family members. Family member group interviews were 52 min on average (range = 30–75 min), with the group interview that included six family members being the longest. In total, eight family members participated, all of whom identified as women, with seven who identified as White, and one who identified as Hispanic. They lived across rural, town, and suburban locations. Three had associate degrees, and five had bachelor’s degrees. Two caregivers were bilingual (i.e., English/Spanish, English/American Sign Language). The participants’ children included two girls and six boys who had a variety of diagnoses (e.g., autism spectrum disorder, down syndrome, visual impairment, hearing impairment, genetic disorder, apraxia), and used a variety of AAC systems (e.g., Proloquo2Go, TD Snap, LAMP Words for Life).

### 5.2. Interview Procedures

Interview participants were asked to review the digital learning platform and materials for the FamTAM Intervention for their respective roles (i.e., SLPs reviewed materials for SLPs, and family members reviewed materials for family members) prior to the online interview. Two members of the research team facilitated each interview and ensured active participation through regular reminders to participants who were less active. The first author led all interviews, and a second team member took field notes. During the interviews, participants were asked to discuss (a) areas in the training and/or coaching materials that they had difficulty understanding, (b) concerns about the use of technology with the online material, and (c) their perceptions of the content, format, technology requirements, and feasibility. Interviews were recorded for later transcription and analysis. Interview participants were compensated with a $50 gift card. See the Supplemental Materials for the interview protocol (S2). To ensure trustworthiness of the data, during interviews, we gathered field notes. We also conducted member checks to verify the accuracy of our data with participants. Interview summaries were sent to all participants via email. They were asked to provide any additional details or clarifications to the summary. Those additional suggestions were incorporated into our findings.

Interview data, including transcripts and field notes, were analyzed using a qualitative content analysis approach (Saldaña, 2021). During content analysis, two members of the research team independently analyzed all data, identified the relevant feedback indicating which aspects of the training were helpful and which needed adjustment, and then shared their findings with the team. Each comment in the interview data, transcripts, and field notes that indicated a helpful aspect of the training or an area needing adjustment was marked and categorized (e.g., video examples were helpful; add additional examples of diverse AAC users). The team then reviewed these categories and engaged in extensive discussion to determine changes needed for the FamTAM Intervention materials based on the data. The team used this to finalize the FamTAM Intervention materials and prepare them for later implementation with families and SLPs.

### 5.3. Interview Findings

Results from our interviews provided valuable insights into adjustments needed to enhance the FamTAM Intervention. From the data, we identified three overarching areas of feedback related to the intervention that were mirrored in both the family member and SLP data. Both family members and SLPs indicated that they viewed the FamTAM Intervention as helpful, but provided suggestions for changes or improvements, and thoughts about the feasibility of implementing the intervention. Specifically, family members felt the real-life examples, content about how to model and the importance of modeling, use of wait time, and preparing were all helpful. They also highlighted the empowering tone and simple language of the materials. A parent said: “There is a general tone of being really encouraging and empowering. I think it’s so essential for overwhelmed parents.” Similarly, SLPs found the video examples, information about why modeling is helpful, and the prepare step to be useful. SLPs also mentioned the coaching information, content aligned for both SLPs and family members, checks for understanding, and structure of the training helpful. Related to the content about coaching, an SLP shared: “When I first learned about coaching it seemed really overwhelming, but [FamTAM] really breaks it down into manageable pieces.”

Despite the mostly positive feedback within interviews, there were numerous suggestions provided. Both SLPs and family members wanted more diverse examples to be added to the content, and an area added within the training that could help participants troubleshoot when things did not go smoothly. A mother suggested: “I think some examples of children of varying abilities ‘cause I know there are some people who use eye tracking.” Related to adding content to support troubleshooting, an SLP suggested: “I feel like anybody who takes a training like this…the very first thing that’s going to come up when you try to implement it is things aren’t going to work…I did think it might be cool if the training…could review challenges that come up.” Because participants in the family interviews only reviewed the materials for the caregivers (not the SLP materials), they also had some suggestions that already aligned with the materials, including materials and guides for SLPs and coaching with families. SLPs also requested immediate feedback on checks for understanding throughout the training modules instead of at the end of each module. Additionally, SLPs requested demonstrations of how to carry out coaching via telepractice.

Overall, both SLPs and caregivers felt that the FamTAM Intervention was feasible, but each group indicated unique challenges that might arise during implementation. SLPs mentioned that the feasibility of implementing the FamTAM Intervention would depend in part on their caseload, administrator support, time in their schedule, and IEPs for consultation, as well as family factors. One SLP shared: “Conceptually I think it’s a great idea, and I think it would be feasible in a lot of situations, but it’s going to depend on my caseload.” Another SLP said: “My current position is an AAC specialist. I have a lot of consultation minutes. The problem for other SLPs is that they don’t have consultation minutes. They only have direct service minutes. So that is a whole thing that would have to change, either doing an amendment or waiting for the next IEP.” Family members felt that implementing the FamTAM Intervention would be challenging if SLPs lacked knowledge in AAC or if there was high SLP turnover. One family member shared: “There’s a lot of SLPs who don’t know a lot about AAC…I feel like that’s a bit of a challenge.”

### 5.4. Adjustments Based on Interviews

After all interviews were complete, adjustments were made to the materials based on the feedback we received from SLPs and family members. The adjustments focused primarily on addressing their concerns about troubleshooting when things went wrong and adding coaching demonstrations. Within the materials, we added video examples of coaching, as well as a section for frequently asked questions that addressed common questions and concerns to assist SLPs and family members in implementing the intervention. While additional diverse examples were requested, including video examples of children who used eye-gaze-based AAC systems, due to a lack of access to these videos, we planned to add them in the final materials with permission from potential participants who represented those examples of diversity. Based on the findings related to feasibility, we also made additional adjustments to our recruitment materials and initial meetings with schools to help schools identify how the intervention might be best implemented in their setting. This included flexibility in coaching approaches, including virtual, in person, and phone/text approaches. After these adjustments were completed, we launched our first tests of the FamTAM Intervention. Based on the performance of our participants in those studies, we will incorporate additional adjustments in the final FamTAM Intervention (see Figure 1).

## 6. Conclusions

As a team, we learned numerous lessons through the iterative process of developing the FamTAM Intervention for SLPs and family members. Engaging in multiple cycles of feedback from both experts and intended users, school-based SLPs and family members, was critical in shaping an intervention that can be used by SLPs and family members in everyday practice. The process emphasizes our commitment to an intervention grounded in current evidence-based practice, but also in practical feasibility. While there remain some logistical barriers that might make implementation difficult (i.e., high SLP caseloads, limited time for coaching, family member overload with therapies and care of children who use aided AAC), the iterative process and multiple perspectives allowed us to address most of the concerns and suggestions raised by the experts, SLPs, and family members, and helped us adjust our recruitment process to ensure a match between the intervention and the schools in which we will engage in future research.

One of the most impactful suggestions emerging from the interviews with family members and SLPs was the creation of frequently asked questions to support SLP and family teams in implementing the FamTAM Intervention even when challenges arise. This has become a living document that is added to as the materials are used by families and SLPs. When concerns arise, we provide new items to help future teams navigate intervention implementation more seamlessly. This dynamic responsive approach reflects our commitment to continued improvement and supporting real-world implementation.

Although we designed this iterative process specifically for the creation of the FamTAM Intervention, the process could help inform other researchers as they develop interventions designed to support children with disabilities, their families, and the professionals who serve them. The process of gathering input from both experts and intervention-interested parties, as well as a person-based approach, has been used in other intervention development applications (Bagawan et al., in press; Douglas et al., under review-a; Terol et al., 2024; Wainer et al., 2018). Additionally, the iterative process we described has been suggested as part of intervention development (Kirchner et al., 2019) and may be particularly useful in implementation since the process of development is transparent (Kirchner et al., 2019; Powell et al., 2019).

## 7. Limitations

As with any project, there were some limitations to note within our development process. First, we had a limited number of participants who helped inform our iterative development process and were recruited primarily through convenience sampling, which may have introduced bias into our data. Second, our expert reviewers were all women and had limited diversity (one who identified as Hispanic). However, these individuals are representative of the current demographics within the field related to this work and were identified because of their expertise and knowledge in all areas related to the intervention. Additionally, despite efforts to recruit diverse SLPs and family members and success recruiting diverse participants during the screening process, the majority of our interview participants were White, with limited diversity represented. We experienced difficulty getting responses and attendance from additional diverse participants during the interview scheduling. This limitation should be considered when implementing the intervention with diverse individuals and may indicate a need to make additional adjustments for diverse families. Additionally, we would like to acknowledge the digital nature of the intervention. Although coaching is provided by SLPs, the digital aspects of training may serve as a barrier for some families. The focus of this article is the development of the FamTAM Intervention. As such, our data include perceptions of our expert reviewers, SLPs, and family members rather than data on direct implementation (e.g., fidelity of coaching by SLPs, fidelity of ALM implementation by family members, child communication data). We intend to report data on direct implementation in future publications related to this project.

## 8. Implications for Future Development

Our next phase of the project includes iterative implementation of the FamTAM Intervention to ensure high-fidelity coaching by SLPs and high-fidelity implementation by family members. Specifically, we have a series of single-case design studies planned to systematically evaluate the coaching fidelity by SLPs and implementation by families. We will also measure outcomes of the intervention for children who use AAC in these studies. These implementation studies will allow us to examine how the intervention functions in authentic contexts, identify any remaining barriers, and refine the materials accordingly. Findings from these series of studies will guide the final adjustments to the FamTAM Intervention and complete our iterative development process. It will also allow us to identify any barriers that might prevent adoption of the intervention or continued feasibility concerns. This also positions us to examine questions related to scalability, sustainability, and long-term adoption, which are essential for broader dissemination.

## 9. Implications for Practice

This project produced the FamTAM Intervention, including professional development for SLPs to support coaching and training materials for family members to support implementation. We anticipate that the online format of the training will provide an accessible and sustainable way to deliver professional development/training and implement the intervention. Additionally, the cascading or train-the-trainer model has a multiplying effect, allowing SLPs to build capacity which can impact numerous children, and families to build capacity to carryover skills in home settings. Beyond supporting families to implement ALM, this intervention may also serve to support school-based SLPs in their work with families.

## Figures and Tables

**Table 1:** FamTAM Intervention components.

Instructional Component	Professional Development Materials for SLPs	Training/Coaching Materials for Caregivers
Overview	Online Training Introduction to FamTAM Intervention FamTAM Intervention Manual Digital manual with step-by-step procedures, instructions, checklists, and supplemental materials	Online Training Introduction to Prepare–Show–Wait–Respond ALM strategy
Description	Online Training Family-Centered Practices/Family Professional Partnership Adult Learning Theory Coaching Practices Prepare–Show–Wait–Respond ALM strategy Data-Based Decision Making	Online Training Prepare Show Wait Respond Summary of Prepare–Show–Wait–Respond ALM strategy
Demonstration	Video examples within training	Video examples within training
Feedback	Checks for understanding within the digital learning platform Discussion board for Q&A within the digital learning platform	Telepractice coaching by SLPs Pre-observation Observation Post-observation

**Table 2:** Expert review feedback and adjustments.

Recommended Adjustments	Representative Quote	Adjustments Made
Clarify module structure	*Might be clearer to integrate modules and split across phases.*	Improved organization and flow
Sequence content for better flow	*Starting with coaching specifics before the fidelity checklist may create easier flow.*	
Emphasize reflection	*Feedback should focus on the coach’s self-reflection and self-selected goals.*	Enhanced content
Use flexible wait time	*Wait at least 5 s… may not be enough for children with motor impairments.*	
Improve slide’s design	*With some minor adjustments, the slides can be much more impactful… fonts and colors that make it easier to read.*	Improved design
Reduce text and add visuals	*Use much less text… rely more on graphics and small icons.*	
Add examples	*Add specific examples of scripts and videos rather than referring participants to external resources.*	Added examples
Add practical examples	*May benefit from more depth/clarification or additional practical examples.*	
Provide routine-based parent examples	*Give parents examples of routine activities where they can model target vocabulary.*	
Strengthen cultural diversity content	*References to cultural diversity felt a bit tangential…a more explicit approach would enrich the content.*	Strengthened cultural content
Include cultural resources	*Many cultural nuances… helpful to include resources on that aspect.*	
Include resources on routines categories	*Include resources like the Family Routines Categories… to reflect on home routines.*	Added resources
Add annotated resource descriptions	*Helpful to have information about each resource, like an annotated bibliography.*	
Use more engaging supplemental materials	*Journal articles alone may not be the best way to engage participants.*	

## Data Availability

The data that support the findings of this study are available from the corresponding authors upon reasonable request due to privacy.

## Supplementary Materials

The following supporting information can be downloaded at: https://www.mdpi.com/article/10.3390/bs16091571/s1, S1: Expert Review Instructions; S2: Interview Protocol. Reference (Krueger & Casey, 2015) is cited in the supplementary materials.
