# Supplementary material for: Development of FamTAM Intervention for Speech and Language Pathologists and Family Members
Source: Behav Sci (Basel). 2026 Sep 4;16(9):1571. doi: 10.3390/bs16091571 (PMC13603157; doi:10.3390/bs16091571)
Supplement: Supplementary file 1 [file behavsci-16-01571-s001.zip › behavsci-4390969-supplementary.pdf]

Supplementary Materials

S1: Expert Review Instructions

As an expert reviewer you will be providing critical feedback along with our SLP and family member interviews at key points in the project. Your review will be iterative with new versions provided for review frequently in the first year of the project.

We are excited to share the first version of our training content with you. This first review cycle will focus only on the training content.

The training materials include PowerPoints that will be presented to participants as recorded videos with accessibility features embedded. For this round of material review we provide the slides with the script that will become the voiceover. We also have a few videos (links) on slides as well as checks for understanding (in the final materials participants will get feedback and correct answers) and resources that will be included within most sections of training. The training includes:

1. Unit 1: FamTAM Training Introduction for SLPs (SLPs only) [LINK]
    a. Module 1- Introduction to FamTAM
    b. Module 2- Partnering and Collaborating with Families
    c. Module 3- Coaching
    d. Checks for understanding
    e. Resources
2. Unit 1: FamTAM Training Introduction for Families (Family Members only) [LINK]
    a. Module 1- Introduction to FamTAm
3. Unit 2: FamTAM Training for Family Members and SLPs (SLPs and Family Members) [LINK]
    a. Module 1- Introduction to Communication and AAC
    b. Module 2- ALM
    c. Module 3- Application of ALM
    d. Checks for understanding
    e. Resources
4. Unit 3: FamTAM Coaching for SLPs (SLPs only) [LINK]
    a. Module 1- ALM Coaching
    b. Module 2- Coaching Application
    c. Module 3- Next Steps
    d. Checks for understanding
    e. Resources

We are requesting that you provide general and specific feedback about the content of the training:

1. Review the slides and script of each PowerPoint/module and add *specific feedback* by adding your comments to the Google slides. You will be able to see comments from other advisory board members. Feel free to interact with their comments.
2. Complete the form [LINK] to provide *general feedback*. Your comments in this form will not be shared with other advisory board members. We provide the content of the Google form below for your reference.

<u>Form Questions for General Feedback</u>:

Thank you for providing *<u>general feedback</u>* about the first draft of the training materials.

1. **E-mail**
2. **Full Name**
3. **Unit I: FamTAM Training Introduction (Families and SLPs)**
   Scale for a-f: *Strongly Agree, Agree, Disagree, Strongly Disagree*
   a. The content of the unit aligns with best practices/recommendations in the literature.
   b. The content in the unit is clear and includes sufficient examples/descriptions.
   c. The videos in the unit are clear and helpful.
   d. The images in the unit represent diverse populations.
   e. The checks for understanding were appropriate.
   f. The resources provided were appropriate.
   g. General feedback about the unit and adjustments/changes that could be made to improve the unit. *Open ended*
4. **Unit II: FamTAM Training for Family Members and SLPs**
   Scale for a-f: *Strongly Agree, Agree, Disagree, Strongly Disagree*
   a. The content of the unit aligns with best practices/recommendations in the literature.
   b. The content in the unit is clear and includes sufficient examples/descriptions.
   c. The videos in the unit are clear and helpful.
   d. The images in the unit represent diverse populations.
   e. The checks for understanding were appropriate.
   f. The resources provided were appropriate.
   g. General feedback about the unit and adjustments/changes that could be made to improve the unit. *Open ended*
5. **Unit 3: FamTAM Coaching for SLPs (SLPs only)**
   Scale for a-f: *Strongly Agree, Agree, Disagree, Strongly Disagree*
   a. The content of the unit aligns with best practices/recommendations in the literature.
   b. The content in the unit is clear and includes sufficient examples/descriptions.
   c. The videos in the unit are clear and helpful.
   d. The images in the unit represent diverse populations.
   e. The checks for understanding were appropriate.
   f. The resources provided were appropriate.
   g. General feedback about the unit and adjustments/changes that could be made to improve the unit. *Open ended*
6. **Do you have any other feedback, comments, or ideas you would like to share with us about the training materials?**

Thank you! We will follow up with any questions we have regarding your feedback.

S2: Interview Protocol

Welcome:

[Instructions before starting the interview]:

Good afternoon and welcome. Thank you for agreeing and taking the time to join our discussion today and to participate in this research project. My name is [xx], and I am a [title] from [xx]. The following team members are assisting me today [xx from xx and xx from xx].

Overview of the topic:

You were all invited here because you are [Speech Language Pathologists/Caregivers] who support children who use AAC. Thank you for completing the online questionnaire that we sent to you and for reviewing the materials we sent. Today, we will be seeking your feedback to inform the development and refinement of our intervention to support young children who use AAC. We appreciate your willingness to share your time and expertise.

Ground rules: *[Krueger & Casey, 2015, p. 118]*

Before we start, I want to review the process for today. During this session, I will ask questions and ask you to respond. There are no wrong answers. We expect that you will have different experiences and points of view. Please share your point of view even if it differs from others who might be present.

Also, for the purposes of capturing everything that is shared, we will record the session for later transcription. Any identifiable information you provide will be removed. We will share a summary of what we learned with you in a couple of weeks to make sure we captured everything correctly and see if you have anything additional you would like to add. After that we will send you a $100 gift card.

The interview will last approximately 45 minutes. We have names displayed in zoom to help us and help you, but feel free to change your name if you prefer. We are interested in hearing from each of you, but don't feel like you have to respond to all of the questions. If you want to follow up on something someone said, give an example, or agree/disagree, please feel free to do so. I am here to ask questions, listen, and make sure everyone has a chance to share.

You can mute yourself unless you want to talk. Do you have any questions before we begin? *[Answer any questions]*

Opening/ice breaker questions for SLP Interview:

Okay, great! Let's get started…Tell us your name, a little about yourself, and one thing you love about working with students who use AAC.

Transition question	1. Describe the students you've supported who use AAC.
Key questions	2. What did you like best about the training materials?
	3. What areas of the training materials need improvement?

	4. Do you feel this intervention would be feasible to carry out with caregivers given your current responsibilities?<br>- Why or why not?<br>- What could be done to make it more feasible?
Ending question	5. Are there any other things we should consider before finalizing the intervention materials?

Opening/ice breaker questions for Caregiver Interview:

Okay, great! Let's get started…Tell us your name, a little about yourself and your child, and one thing you love about your child who uses AAC.

Transition question	1. Tell us about the type of AAC system your child uses and how long they have been using AAC.
Key questions	2. What did you like best about the training materials?
	3. What areas of the training materials need improvement?
	4. Do you feel this intervention would be feasible in your home supported by the school SLP?<br>- Why or why not?<br>- What could be done to make it more feasible?
Ending question	5. Are there any other things we should consider before finalizing the intervention materials?

Closing:

We are still looking for more caregivers and SLPs to provide us with feedback, especially those who are from diverse backgrounds. We want to be sure our intervention is appropriate for a wide variety of SLPs and families. What Facebook groups might be good to post in? Would you be willing to share a flyer to your networks? Thank you so much for participating in our interview today. In a few weeks we will send you a short summary of the session we conducted today to make sure we captured everything correctly and see if you have anything additional you would like to add. After that we will send your gift card to the email you provided.

Again, thank you for your time. Your participation is integral to helping us develop an intervention that can best support family members and young children who use AAC.

Acknowledgements

We would like to thank the expert reviewers and interview participants for the feedback they provided to support the development of the FamTAM Intervention.

Author Contributions

Conceptualization: S.N.D. and H.M.; Methodology: S.N.D. and H.M.; Validation: S.N.D, H.M. and S.D-J.; Formal Analysis: All authors; Investigation: All authors; Resources: S.N.D, H.M., S.D-J., A.B, and A.K.T.; Data Curation: All authors; Writing – Original Draft: S.N.D. and H.M; Writing – Review and Editing: All authors; Visualization: S.N.D.; Supervision: S.N.D, H.M. and S.D-J.; Project Administration: S.N.D, H.M. and S.D-J.; Funding Acquisition: S.N.D. and H.M

References

American Speech-Language-Hearing Association (ASHA) (n.d.). Augmentative and alternative communication.https://www.asha.org/public/speech/disorders/aac/?srsltid=AfmBOopyiMhiGXGAd8mix5uZm8tNWYVWf98GhQjTA8xAhh91n7YfOj1Y

Association for Behavior Analysis International (n.d.). Interprofessional collaborative practice between behavior analysts and speech-language pathologists. https://www.abainternational.org/constituents/practitioners/interprofessional-collaborative-practice.aspx

Author. (in preparation).

Author. (under review a).

Author. (under review b).

Author. (2021).

Author. (2022).

Author. (2023).

Behavior Analyst Certification Board. (2020). *Ethics code for behavior analysts.* https://bacb.com/wp-content/ethics-code-for-behavior-analysts/

Biggs, E. E., Carter, E. W., & Gilson, C. B. (2018*).* Systematic review of interventions involving aided AAC modeling for children with complex communication needs. *American Journal on Intellectual and Developmental Disabilities, 123*(5), 443–473.

https://doi.org/10.1352/1944-7558-123.5.443

Biggs, E. E., Carter, E. W., Bumble, J. L., Barnes, K., & Mazur, E. L. (2018). Enhancing peer network interventions for students with complex communication needs. *Exceptional Children*, 85(1), 66-85. https://doi.org/10.1177/0014402918792899

Bradbury, K., Dennison, L., Little, P., Yardley, L., & Muller, I. (2019). Using mixed methods to develop and evaluate person-based interventions: The person-based approach. *British Journal of Health Psychology, 24*(3), 700–719. https://doi.org/10.1111/bjhp.12360

Coburn, K. L., Jung, S., Ousley, C. L., Sowers, D. J., Wendelken, M., & Wilkinson, K. M. (2021). Centering the family in their system: a framework to promote family-centered AAC services. *Augmentative and Alternative Communication, 37*(4), 229-240. https://doi.org/10.1080/07434618.2021.1991471

Denzin, N.K. (1978). *Sociological methods: A sourcebook.* McGraw-Hill.

Instructure. (2024). Canvas Learning Management System [Software]. https://www.instructure.com/canvas

Kent-Walsh, J., Murza, K. A., Malani, M. D., & Binger, C. (2015). Effects of communication partner instruction on the communication of individuals using AAC: A meta-analysis. *Augmentative and Alternative Communication*, *31*(4), 271-284. https://doi.org/10.3109/07434618.2015.1052153

Kirchner, J. E., Smith, J. L., Powell, B. J., Waltz, T. J., Proctor, E. K. (2019). Getting a clinical innovation into practice: An introduction to implementation strategies. *Psychiatry Research, 283*, 112467. https://www.sciencedirect.com/science/article/pii/S0165178119307413?via%3Dihub

Kummerer, S. E. (2012). Promising strategies for collaborating with Hispanic parents during family-centered speech-language intervention. *Communication Disorders Quarterly*, 33, 84–95. https://doi.org/10.1177/1525740109358453

Lebel, T., Olshtain, E., & Weiss, P. L. (2005). Teaching teachers about augmentative and alternative communication: Opportunities and challenges of a web-based course.

Augmentative and Alternative Communication, 21(4), 264-277. https://doi.org/10.1080/07434610500140311

Lim, C. P. (2004). Engaging learners in online learning environments. *TechTrends, 48*(4), 16-23. https://doi.org/10.1007/BF02763440

Lincoln, Y. S., & Guba, E. G. (1985). *Naturalistic inquiry*. Sage.

Meadan, H., Chung, M. Y., Sands, M. M., & Snodgrass, M. R. (2020). The cascading coaching model for supporting service providers, caregivers, and children. *Journal of Special Education. 54*(2), 113-125. https://doi.org/10.1177/0022466919884070

Meadan, H., Lee, J. D., Sands, M. M., Chung, M. Y., & Garcia-Grau, P. (2022). The coaching fidelity scale (CFS) development and evaluation of an observational measure of coaching fidelity. *Infants and Young Children*, *36*(1), 37-52. https://doi.org/10.1097/IYC.0000000000000231

Meadan, H., Snodgrass, M., Meyer, L., Fisher, K., Chung, M., & Halle, J. (2016). Internet-based parent-implemented intervention for young children with autism: A pilot study. *Journal of Early Intervention*, *38*(1), 3-23. https://doi.org/10.1177/1053815116630327

Pope, L., Light, J., & Laubscher, E. (2025). The effect of naturalistic developmental behavioral interventions and aided AAC on the language development of children on the autism spectrum with minimal speech: A systematic review and meta-analysis. *Journal of Autism and Developmental Disorders*, *55*(9), 3078–3099. https://doi.org/10.1007/s10803-024-06382-7

Powell, B.J., Fernandez, M.E., Williams, N.J., Aarons, G.A., Beidas, R.S., Lewis, C.C., Weiner, B.J., (2019). Enhancing the impact of implementation strategies in health care: A research agenda. *Public Health 7* (3), 1–9. https://doi.org/10.3389/fpubh.2019.00003

Quinn, E. D., Kaiser, A. P., & Ledford, J. R. (2020). Teaching preschoolers with down syndrome using augmentative and alternative communication modeling during small group dialogic reading. *American Journal of Speech-Language Pathology, 29*, 80–100. https://doi.org/10.1044/2019_AJSLP-19-0017

Roberts, M. Y., & Kaiser, A. P. (2011). The effectiveness of parent-implemented language interventions: A meta-analysis. *American Journal of Speech-Language Pathology, 20*, 180–199. https://doi.org/10.1044/1058-0360(2011/10-0055)

Saldaña, J. (2021). *The coding manual for qualitative researchers* (4th ed.). Sage.

Seligman, M., & Darling, R. B. (2017). *Ordinary families, special children: A systems approach to childhood disability*. Guilford Publications.

Smith, A. L. & Barton-Hulsey, A. (2016). AAC and families: Dispelling myths and empowering parents. *Perspectives of the ASHA Special Interest Groups, 12*(1), 10-20. https://doi.org/10.1044/persp1.SIG12.10

Soto, G. (2012). Training partners in AAC in culturally diverse families. *Perspectives on Augmentative and Alternative Communication*, *21*(4), 144-150. https://doi.org/10.1044/aac21.4.144

Sun, T., Bowles, R., Douglas, S. N., Plavnick, J. (2023). Response time of young children with complex communication needs following a communication opportunity. *Exceptional Children, 90*(1), 27-42. https://doi.org/10.1177/00144029221146574

Thiemann-Bourque, K. S., McGuff, S., & Goldstein, H. (2017). Training peer partners to use a speech-generating device with classmates with autism spectrum disorder: Exploring communication outcomes across preschool contexts. *Journal of Speech, Language, and Healing Research, 60,* 2648-2662. https://doi.org/10.1044/2017_JSLHR-L-17-0049

Tong, A., Sainsbury, P., & Craig, J. (2007). Consolidated criteria for reporting qualitative research (COREQ): A 32-item checklist for interviews and focus groups. *International Journal for Quality in Health Care*, *19*, 349-357. https://doi.org/10.1093/intqhc/mzm042

Wainer, A. L., Dvortcsak, A., & Ingersoll, B. (2018). Designing for dissemination: The utility of the deployment-focused model of intervention development and testing for parent mediated intervention. In *Handbook of parent-implemented interventions for very young children with autism* (pp. 425–440). Springer.

Woods, J. J., Wilcox, M. J., Friedman, M., & Murch, T. (2011). Collaborative consultation in natural environments: Strategies to enhance family-centered supports and services. *Language Speech and Hearing Services in Schools, 42*, 379. https://doi.org/10.1044/0161-1461(2011/10-0016)

Yardley, L., Morrison, L., Bradbury, K., & Muller, I. (2015). The person-based approach to intervention development: Application to digital health-related behavior change interventions. *Journal of Medical Internet Research, 17*(1), e30. https://doi.org/10.2196/jmir.4055
